# One-Step Bulk-Suspension Polymerization of Polyethylene Glycol-Based Copolymer Microspheres for Phase Change Textiles

**DOI:** 10.3390/polym15092090

**Published:** 2023-04-27

**Authors:** Guohang Zhou, Jiexiang Zeng, Song Tang, Zijian Bai, Jianyu Jiang, Hong Zhang, Yan Wang

**Affiliations:** School of Textile and Material Engineering, Dalian Polytechnic University, Dalian 116034, China

**Keywords:** phase change microspheres, polyethylene glycol, polystyrene/maleic anhydride, graft copolymerization, phase change textiles

## Abstract

The paper presents a feasible strategy through one-step bulk-suspension polymerization, grafting PEG onto an in situ synthesized copolymer. In more detail, PEG was grafted onto a homemade polystyrene/maleic anhydride copolymer (SMA) via bulk-suspension polymerization with poly(vinyl alcohol) as a suspending agent. According to the optimal reaction conditions, the grafting rate of PEG was 56.2% through chemical titration experiments. At the same time, the quantitative relationship between the grafting rate and enthalpy was demonstrated for the first time in a PEG-based solid–solid phase change material (S-SPCM). Morphology observation revealed that the obtained S-SPCM is made up of white microspheres of approximately 100–150 μm. The powdery product polystyrene/maleic anhydride grafted polyethylene glycol (SMA-g-PEG) obtained through bulk-suspension polymerization endowed that the whole product could be used directly as a phase change material without postprocessing. The melting enthalpy and crystallization enthalpy of SMA-g-PEG were 79.3 J/g and 76.9 J/g, respectively. Based on the effective fixed load of PEG, the macrostructure of SMA-g-PEG was almost unchanged at 70 °C compared with the macrostructures at 20 °C, and the latent heat of SMA-g-PEG was decreased slightly after 1000 thermal cycles. Overall, the obtained SMA-g-PEG can be used as a filler in insulation materials and composited with fibers to obtain phase change thermoregulated smart textiles.

## 1. Introduction

Thermal storage systems and technologies can improve energy efficiency and utilization for energy conservation, environmental protection, and sustainable development [1]. Simultaneously, they can serve as a pivotal factor in ensuring a harmonious balance between energy supply and demand while also contributing to the establishment of a sustainable energy infrastructure. Organic phase change materials (PCMs) possess the characteristics of a polymer in solid formability, stable performance, non-corrosive material, reduced toxicity, and are less prone to subcooling and phase separation [2,3]. Organic PCMs have shown significant potential in various applications, including fabrics, building energy storage, insulated walls, solar energy, and energy storage batteries [4,5], etc. Currently, the research on organic PCMs in thermal storage systems is flourishing to some extent, such as phase change textiles [6,7]. Malaquias et al. [8] added PCMs to fire-protective clothing for their thermal transformation, while Niles et al. [9] used a pad–dry–cure method to coat polyester with sodium-alginate-microencapsulated PEG-1000 for textile performance studies.

Among organic PCMs, PEG is a multifunctional solid–liquid material with high latent heat, low thermal hysteresis effect, and an extensive temperature applicability range [10]. Unfortunately, PEG exhibits poor thermal conductivity and has a tendency to leak during use, which leads to a depletion in the phase change heat storage capacity of natural materials [11]. It, thus, drives a motivation to develop the technology for solid loading to improve the stability of phase change. The various techniques for fixed loading include chemical grafting, physical blending, porous material adsorption, and microcapsule coating [12,13,14], etc. The chemical grafting method, which involves the formation of a covalent bond between the PEG and the organic matrix, has aroused extensive research in the PCM field. This is primarily due to the highly stable PCMs that are formed as a result of this method [15]. For instance, Ahmet Sarı et al. [16] used three different molecular weights of a PEG-grafted styrenic copolymer, producing a novel solid–solid phase change material. The phase transition enthalpies were high (107–155 J/g), and the range of phase transition temperatures was 40–45 °C. However, the use of chloroform as a solvent was dangerous and environmentally unfriendly. Niu Z et al. [17] prepared flexible electro-/photo-driven energy storage polymer fibers by integrating conductive silver nanoflowers and poly(3,4-ethylenedioxythiophene)/poly(styrenesulfonate) as well as hydrophobic fluorocarbon resin. The lantern heat was in a range of 98.6–126.5 J/g. A smart energy storage fiber with integrative properties could be woven into fabrics. Ahmet Sarı et al. [18] synthesized a novel polymeric solid–solid phase change material (S-SPCM). The phase change temperature of S-SPCM was about 40–45 °C and had a relatively high latent heat capacity. However, the cost of using SMA as a raw material is high, and the reaction time is relatively long (24 h). On the other hand, the sample is in lump form and requires post-treatment before use. Therefore, prepared PEG-based S-SPCMs could be directly used in feasible preparation methods, but this remains a challenge.

In this paper, PEG-based S-SPCMs with good phase change properties were prepared through grafting copolymerization using homemade SMA as a substrate and poly(vinyl alcohol) as a suspending agent. The combination of PEG and SMA exhibited exceptional phase change stability. Bulk-suspension polymerization [19] not only shortened the reaction time but also rendered the powder property of the as-prepared product, eliminating the need for post-processing before use. The detailed preparation of SMA-g-PEG and its Fourier-transform infrared spectrometer, X-ray diffraction, POM analysis, and differential scanning calorimetric data were illustrated. The resulting SMA-g-PEG had a clear spherical structure, with latent heat of 79.3 J/g at around 42.09 °C. The grafting rate of PEG measured via chemical titration was 56.2%, and the quantitative relationship between the grafting rate and enthalpy was demonstrated for the first time in PEG-based PCMs. The stable phase change in SMA-g-PEG through this simple synthesis method has great potential applications, such as use as a filler in building thermal insulation materials [20,21] and composited with fibers to obtain phase change thermoregulated smart textiles [22], which could realize instant energy conversion/storage, temperature regulation, and photovoltaic/thermal air collectors [23,24].

## 2. Experimental

### 2.1. Chemicals

Polyethylene glycol (PEG) (M_w_: 2000 g/mol) was purchased from Sinopharm Chemical Reagent Co., Ltd., Shanghai, China. Styrene and 1,2-dimethylbenzene were provided by Tianjin Damao Chemical Reagent Factory, China. Maleic anhydride (MAH), poly(vinyl alcohol) (PVA), 2-methylpropionitrile (AIBN), potassium hydroxide (KOH), phenolphthalein, and potassium hydrogen phthalate were supplied by Maclean Biochemical Co., Ltd., Shanghai, China. Isopropanol, concentrated hydrochloric acid, acetone, and pyridine were obtained from Tianjin Komiou Chemical Reagent Co., Ltd., Tianjin, China. Anhydrous ethanol was acquired from Tianjin Fuyu Fine Chemical Co., Ltd., Tianjin, China.

### 2.2. Synthesis of SMA-g-PEG Microspheres

**Figure 1:**
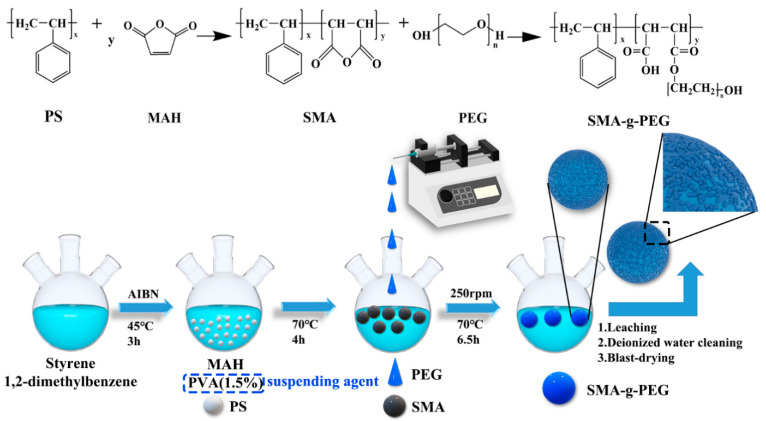
Reaction schema regarding the synthesis of the SMA-g-PEG gel microspheres.

The synthetic route of SMA-g-PEG is illustrated in Figure 1. Styrene (13.2 mL), 1,2-dimethylbenzene (1.32 mL), and AIBN (0.8 g) were added to a three-necked flask at 45 °C for 3 h. Then, 2 g of MAH was added in polystyrene (PS) oil beads at 70 °C for 2 h. When MAH was completely reacted, 1.5% concentration solution of PVA (20 mL) was added under violent stirring at 70 °C for 2 h to form an oil phase solution [19,25]. Once the SMA copolymerization reaction was completed, the PEG aqueous solution (2%, 50 mL) of 70 °C was added to the three-necked flask at a constant rate, and the stirring speed was adjusted to 250 rpm for 6.5 h. In the end, the obtained solution was filtered, washed three times repeatedly with deionized water, and dried for 24 h. Herein, the obtained PEG-based PCMs are referred to as SMA-g-PEG, which are denoted as the solid–solid phase change materials (S-SPCM) via chemical reactions and physical cross-linking mechanisms. In more detail, grafting long PEG chains in SMA-g-PEG results in physical cross-linking through inter-chain physical entanglement and hydrogen bonding between SMA-g-PEG molecules. Parameters for the synthesis experiment were selected according to the previous experimental results [16]. The optimum process was carried out using the performance of phase change as a benchmark, with the specific variables and sample numbers, as shown in Table 1.

### 2.3. Characterization of SMA-g-PEG

The FTIR spectra of the as-prepared PS (polystyrene), PEG, SMA, and SMA-g-PEG were taken on a KBr disk in a wave number range of 4000–400 cm^−1^ using Fourier-transform infrared spectrometer (FTIR) (Spectrum One-B, Perkin Elmer Co., Ltd., Waltham, MA, USA). The surface morphology of SMA-g-PEG was studied using a scanning electron microscope (SEM) (JSM-7800F, Japan Electronics Co., Ltd., Tokyo, Japan). X-ray diffraction (XRD) was used to determine the crystalline nature of the samples (D/max-3B, Rigaku Corporation Co., Ltd., Tokyo, Japan) (scanning voltage: 40 kV, scanning angle: 5~70°, scanning speed: 5°/min). The phase change properties of SMA-g-PEG used differential scanning calorimetry (DSC) (Q2000, TA Instruments, New Castle, DE, USA) (sample mass: 5–10 mg, N_2_, N_2_ flow rate: 10 mL/min, test conditions: the heating or cooling rate was 10 °C/min between 0 and 100 °C.). The thermal stability of SMA-g-PEG was measured using thermogravimetric analysis (TGA) (Q50, TA Instruments, New Castle, DE, USA) (sample mass: 5–10 mg, N_2_, N_2_ flow rate: 50 mL/min, test conditions: heating rate was 20 °C/min from 25 to 600 °C.). The crystalline morphology was observed with a polarizing microscope (POM) (BX51, Olympus, Tokyo, Japan) (heat up the hotplate to 100 °C and hold for 2 min). The thermal cycle test used a thermal cycler (MiniAmp, Thermo Fisher, Waltham, MA, USA) (heating at 70 °C for 30 min and cooling at 20 °C for 30 min, heating/cooling rate: 10 °C/min). Infrared thermography imaging was performed with infrared imaging pyrometer (TG165, FLIR Systems Inc, Wilsonville, OR, USA). Temperature recorder was used for recording temperature changes (Tlog 10EH, Jingchuang Electric, Xuzhou, China) (heating at 70 °C and cooling at 20 °C).

### 2.4. Determination of PEG Grafting Rate

The grafting rate of PEG in SMA-g-PEG was determined through titration experiments repeated three times to obtain the average value for calculating the grafting rate.

Phenolphthalein, 0.3 mol/L KOH-ethanol, and HCl-isopropanol solutions were prepared as standards for the titration experiments [26,27].

Next, the graft was refined: the sample was dried at 90 °C for 12 h; then, 5 g was added into a flask containing 40 mL of 1,2-dimethylbenzene and dissolved by heating at reflux. Once completely dissolved, it was poured into a large amount of acetone while hot then left to cool, filtered, and precipitated. In the end, the precipitate was washed twice with acetone, and the flocculent was dried under vacuum at 90 °C for 24 h.

Then, the graft titration was as follows: 0.5 g of refined SMA-g-PEG and 80 mL of 1,2-dimethylbenzene were added into a three-necked distillation flask, which was refluxed at 90 °C for 10 min to dissolve the sample. Afterward, a drop of water and pyridine and 1.5 mL of KOH-ethanol standard solution were added and refluxed at 100 °C for 30 min. Then, isopropanol (6 mL) and phenolphthalein reagent (two drops) were added and stirred with magnetic force to obtain a dark-red solution. The titration was stopped when the solution was pink, and the volume of HCl-isopropanol solution consumed was recorded *V*. After the titration was completed, a back-titration test was carried out with KOH-ethanol solution.

Finally, the ingraft titration was as follows: 0.5 g of SMA was added into a three-necked flask. The above operations were repeated, the volume of HCl-isopropanol marker *V_x_* consumed by the test was noted, and the volume of KOH-ethanol marker Δ*V* covered by the system was calculated. The formula is as follows:

(1) $$\Delta V=\left(1.5\times C_{1}-V_{X}\times C_{2}\right)/C_{1\;}$$

The grafting rate is calculated using

(2) $$C=1500\times \frac{\left[C_{1}\times \left(V_{1}-\Delta V\right)-V_{2}\times C_{2}\right]}{2m}$$

where *V*_1_ and *V*_2_ are the volume (mL) of KOH-ethanol marker and HCl-isopropanol marker, *C*_1_ and *C*_2_ are the concentration (mol/L) of KOH-ethanol marker and HCl-isopropanol marker, and *m* is the mass (g) of material.

### 2.5. Process Performance

Based on the DSC results, process performance parameters (including the yield and grafting rate) were estimated.

The yield is calculated as follows:

(3) $$\mathrm{yield}=\frac{\mathrm{mass}\;\mathrm{of}\;\mathrm{SMA}-g-\mathrm{PEG}}{\mathrm{The}\;\mathrm{total}\;\mathrm{mass}\;\mathrm{of}\;\mathrm{PEG}\;\mathrm{and}\;\mathrm{monomers}\;\mathrm{used}}\times 100\%$$

The grafting rate is calculated as follows:

(4) $$\mathrm{grafting}\;\mathrm{rate}=\frac{\Delta H_{\mathrm{SMA}-g-\mathrm{PEG}}\;}{\Delta H_{\mathrm{Pure}\;\mathrm{PEG}}}\times 100\%$$

where ∆H_SMA-g-PEG_ is the latent heat of SMA-g-PEG (J/g) and ∆H_Pure PEG_ is the latent heat of pure PEG (J/g).

## 3. Results and Discussion

### 3.1. Factors Influencing the Phase Change Properties of SMA-g-PEG

**Figure 2:**
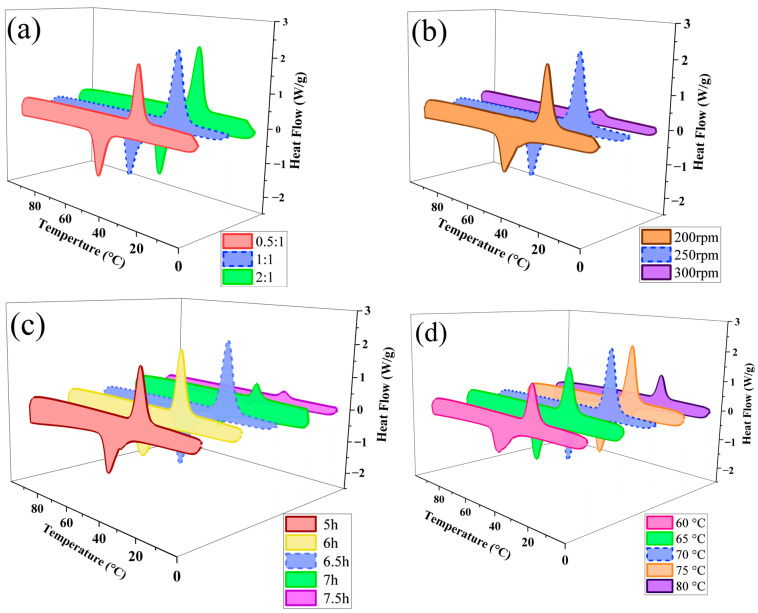
DSC curves of the relationship between phase change behavior and variables. (**a**) Proportions of PS: PEG (mol: mol). (**b**) Stirring rates. (**c**) Reaction time. (**d**) Reaction temperatures.

The DSC curves of SMA-g-PEG under various conditions are shown in Figure 2, and the corresponding data are listed in Table 2. The grafting and melting values of SMA-g-PEG microspheres were not always homogeneous, and there was a sampling error in the DSC test. In the A sample studies, the highest latent heat and yield were 79.3 J/g and 54.1% for the sample with PS: PEG = 1:1 (mol: mol). For the sample with PS: PEG = 0.5:1 (mol: mol), the PEG was not grafted fully with low PS content. The excess PEG was not solidly loaded, and it would be lost during the post-treatment process, resulting in a reduction in the latent heat. As PS was in excess (PS: PEG = 2:1 (mol: mol)), the relative content of PEG was decreased, making the grafting of PEG inadequate and leading to a reduction in latent heat. Based on optimum enthalpy and yield, the raw material proportion of 1:1 was chosen to be the best.

As shown in Figure 2b, SMA-g-PEG at a stirring rate of 250 rpm was the highest latent heat sample (79.3 J/g) in the series of B samples. If the stirring rate was too low, the contact between SMA and PEG molecules in the reaction system was limited, and it was difficult for the grafting reactions to occur [26]. Meanwhile, the grafting reaction became slower, and the grafting rate decreased, resulting in the reaction being unable to finish within the set time [28]. On the other hand, a high stirring rate reduced the covalent bond formation between SMA and PEG, which led to an implosion of the reaction system with rapidly increased viscosity and difficulties in heat dissipation and stirring, resulting in a lower grafting rate [29]. Therefore, a stirring rate of 250 rpm was chosen as the optimum process.

DSC curves of C samples prepared with different reaction times are illustrated in Figure 2c. As shown in Table 2, the highest latent heat (79.3 J/g) was obtained for the sample prepared via a reaction for 6.5 h. While the reaction time was short, the grafting reaction did not easily occur sufficiently, resulting in a lower latent heat. A long reaction time made excessive PEG grafting on SMA form entanglement, which was detrimental to the crystallization of PEG. Further, the product easily formed an entangled lumpy structure, which led to a longer experimental period, higher production costs, and energy waste in practical applications. Therefore, a reaction time of 6.5 h was chosen as the optimum process.

The DSC curves of sample D prepared at different reaction temperatures are shown in Figure 2d. The sample prepared at 70 °C obtained the highest latent heat of 79.3 J/g, as shown in Table 2. The lower the reaction temperature, the lower the efficiency of the initiator AIBN [28], resulting in a slower reaction rate and lower latent heat. The reaction rate was accelerated with a high reaction temperature, and the unreacted MAH was dissolved in the solution. The microspheres stuck together and lumped easily, making the phase change performance of PEG worse with lower latent heat.

In summary, at a raw material ratio of 1:1, a reaction time of 6.5 h, a reaction temperature of 70 °C, and a stirring rate of 250 rpm, the optimum reaction conditions for SMA-g-PEG preparation were obtained.

The average grafting rate of PEG was 56.2% through chemical titration experiments. According to the ratio of the latent heat of SMA-g-PEG (79.3 J/g) and the latent heat of PEG (151.1 J/g) (Table 3), the grafting rate was 52.5% in theoretical value. Obviously, the phase change enthalpy can be roughly estimated via chemical titration, whereby the quantitative relationship between grafting rate and enthalpy is demonstrated for the first time in PEG-based PCMs. In addition, the difference between the theoretical value and the titration value was due to the fact that PEG crystallization in the PCMs could not achieve the ideal degree of regularity as in the pure PEG because of chain end curl, wafer thickness, crystallization size, and crystallization degree [30], etc. On the other hand, the phase transition temperature and enthalpy were reduced with a high grafting rate. Because the covalent bonding is much greater than the van der Waals forces and hydrogen bonding in the case of blending, the movement of polymer chains is greatly inhibited after graft polymerization, which has great impacts on phase transition behavior [22,31]. According to the optimal reaction conditions, the grafting rate of SMA-g-PEG was 56.2%. On the other hand, the average grafting efficiency of PEG was 47.3%, indicating the high availability of PEG. Overall, one-step bulk-suspension polymerization of grafting PEG onto the in situ synthesized copolymer was a feasible method for the PEG-based PCM.

### 3.2. Structure and Morphology Analysis of SMA-g-PEG Microsphere

**Figure 3:**
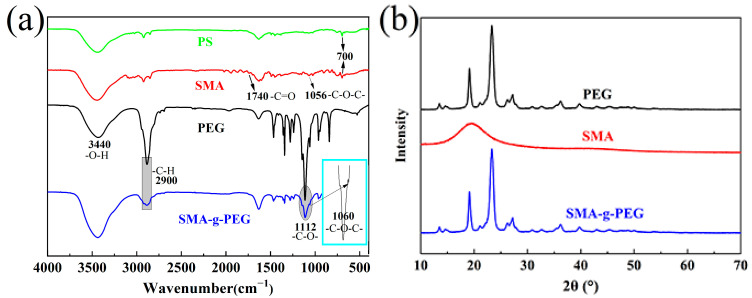
(**a**) FTIR spectra of PS, SMA, PEG, and SMA-g-PEG. (**b**) XRD curves of PEG, SMA, and SMA-g-PEG.

FTIR observation was conducted to inspect the characteristic peak of the characteristic groups in the SMA and SMA-g-PEG, in which the copolymerization reaction and graft polymerization were identified (Figure 3a). The characteristic peaks of SMA at 1056 cm^−1^, 1740 cm^−1^_,_ and 700 cm^−1^ [18], representing the stretching vibration peak of C–O–C, C=O, and C–H (benzene ring), indicated that the copolymerization reaction of PS and MAH proceeded successfully. When SMA was grafted by PEG, the peak at 1112 cm^−1^ and 2900 cm^−1^ of C–O and C-H (stretching vibration) of PEG shifted slightly, and the area of characteristic peaks decreased in SMA-g-PEG, which confirms the grafting reaction. Additionally, the peaks of C–O–C at 1056 cm^−1^ (symmetric stretching vibrations) were not decreased for SMA, which may be attributed to the newly appearing band at 1060 cm^−1^, representing the stretching vibration peaks of C–O–C.

The XRD spectra of PEG, SMA, and SMA-g-PEG are given in Figure 3b. There were almost no crystallization peaks in SMA, demonstrating that all the crystals of SMA-g-PEG should be inherited from PEG. Meanwhile, the 2θ positions of SMA-g-PEG were exactly the same characteristic peaks as PEG (19.2°, 23.3°, and 26.9°) [32]. Although the intensity of the crystalline peak was reduced slightly compared to PEG, the crystal structure of PEG in SMA-g-PEG was almost exactly the same as that of the pristine PEG. Therefore, the PEG in SMA-g-PEG still possessed good crystallization ability, endowing excellent phase change properties.

**Figure 4:**
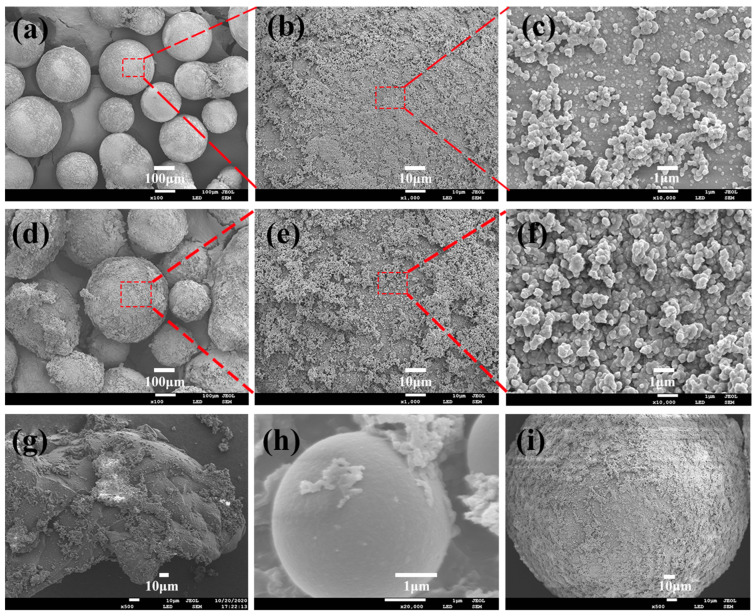
SEM images of sample A SMA-g-PEG (**a**) ×100, (**b**) ×1000, and (**c**) ×10,000 (PS: PEG = 1:1 (mol: mol)); sample A SMA-g-PEG (**d**) ×100, (**e**) ×1000, and (**f**) ×10,000 (PS: PEG = 0.5:1 (mol: mol)); (**g**) sample B SMA-g-PEG (300 rpm); (**h**) sample C SMA-g-PEG (7.5 h); (**i**) SMA-g-PEG after 1000th thermal cycle.

Figure 4 shows SEM images of the SMA-g-PEG particles. It could be observed that SMA-g-PEG (sample A: PS: PEG = 1:1 (mol: mol)) presents a spherical structure, and the particle size of the microspheres is approximately in a range of 100–150 μm. The overall appearance of the SMA-g-PEG particles was smooth, and the graft of PEG occurred outside of SMA (Figure 4a), on the surface of which the grafting was evenly distributed (Figure 4b,c) and no holes appeared after the leaching treatment. In Figure 4d, it can be seen that the surface of the SMA-g-PEG particles was rough due to an increase in PEG content. The inhomogeneous distribution of PEG (Figure 4e,f) led to certain changes in the morphology of the microspheres and a reduction in latent heat. As shown in Figure 4g, the morphology of the SMA-g-PEG was affected by the high stirring rate, and the shape became irregular, which led to a decrease in latent heat. A long reaction time would make excessive PEG grafting on SMA form entanglement (Figure 4h), which is detrimental to the crystallization of PEG. On the other hand, SMA-g-PEG was still morphologically stable after the 1000th thermal cycle (Figure 4i).

**Figure 5:**
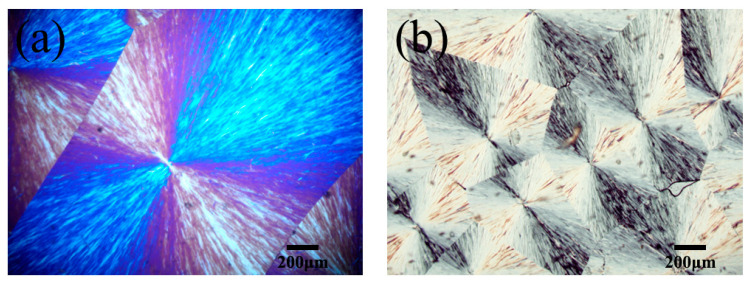
POM images of (**a**) PEG and (**b**) SMA-g-PEG at 25 °C.

The POM images of PEG and SMA-g-PEG are shown in Figure 5. PEG presented typical spherulite structures with a “black cross extinction” character at 25 °C (Figure 5a), and SMA-g-PEG retained the same features (Figure 5b). Although the spherulite size of SMA-g-PEG was smaller than that of PEG, the crystallization ability was adequate. The notable spherulite affirmed the successful grafting of PEG in SMA-g-PEG, which further endowed the excellent phase change properties.

### 3.3. TGA Analysis of SMA-g-PEG

**Figure 6:**
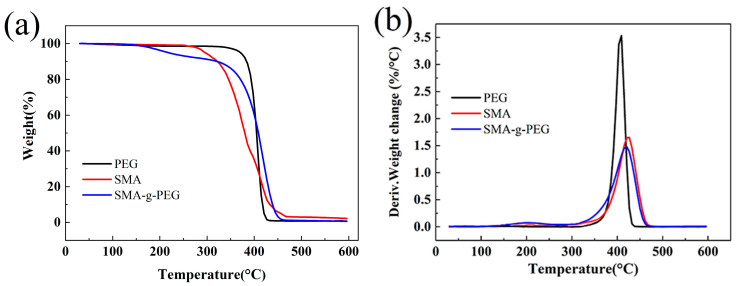
(**a**) TG curve, (**b**) DTG curve for PEG and SMA-g-PEG.

TG and DTG curves of PEG, SMA, and SMA-g-PEG are displayed in Figure 6. It could be seen that the decomposition of SMA-g-PEG started at 340.2 °C and reached the highest weight loss rate at 402.2 °C. In Figure 6a, the 3.7% thermal weight loss before 200 °C was mainly due to thermal decomposition of PVA on the surface of the copolymer, which was not completely washed off [33], and 5.3% thermal weight loss in a range of 200–320 °C was mainly due to the thermal decomposition of the anhydride group (in SMA) to carbon dioxide [34,35]. In Figure 6b, the decomposition of SMA started at 280.8 °C and reached the highest weight loss rate (3.5%/°C) at 400.1 °C. The decomposition of PEG started at 333.4 °C and reached the highest weight loss rate (1.5%/°C) at 426.7 °C. It was not significantly different between PEG and SMA-g-PEG in terms of the starting decomposition temperature and highest weight loss rate. However, the final decomposition temperature of SMA-g-PEG (454.4 °C) was 28.4 °C higher than that of PEG (426.0 °C), indicating that the grafting of PEG improved the thermal stability of SMA-g-PEG. On the other hand, the combination of chemical grafting and physical cross-linking through hydrogen bonding contributed to the improved thermal stability of SMA-g-PEG gel microspheres.

### 3.4. Thermal Reliability of SMA-g-PEG

**Figure 7:**
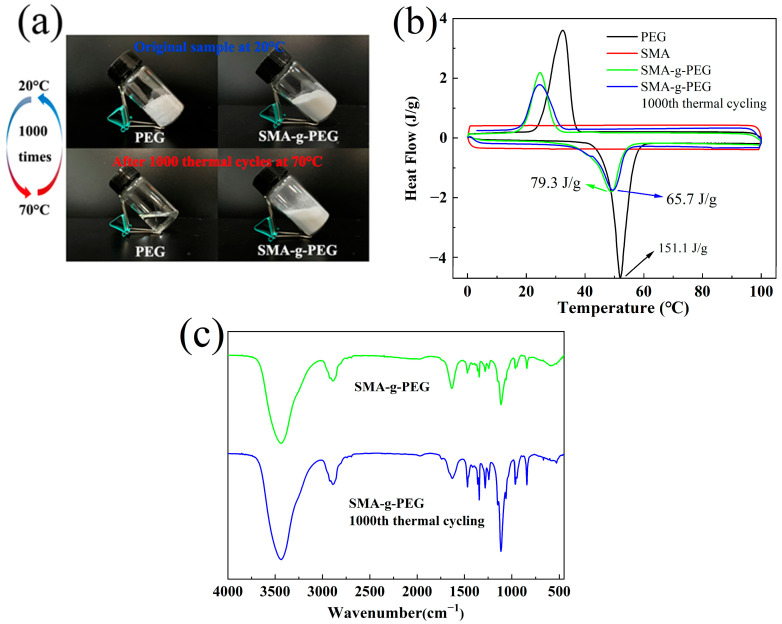
(**a**) Digital photos of PEG and SMA-g-PEG from 20 °C to 70 °C after 1000 thermal cycles, (**b**) DSC curves of PEG, SMA, SMA-g-PEG and SMA-g-PEG after 1000th thermal cycle, (**c**) FTIR spectra of SMA-g-PEG and SMA-g-PEG after 1000th thermal cycle.

The digital photos of PEG and SMA-g-PEG, which were heated from 20 °C to 70 °C and cooled to 20 °C 1000 times, are shown in Figure 7a. The macroscopic morphology of SMA-g-PEG remained relatively unchanged and continued to exist in a powdered state after 1000 heating–cooling cycles, which indicated that the phase change performance of SMA-g-PEG was stable and PEG did not leak. The DSC curves and data for SMA-g-PEG and after the 1000th thermal cycle are displayed in Figure 7b and Table 3. Compared to the as-prepared SMA-g-PEG, the melting enthalpy of SMA-g-PEG after thermal cycling was slightly decreased from 79.3 to 65.7 J/g, and slightly shifted peak melting and solidifying temperatures occurred. Degradation of PEG may occur after the 1000th thermal cycle at 70 °C due to the time–temperature equivalence phenomenon for polymers [36], resulting in a decrease in lantern heat. The FT-IR spectra are shown in Figure 7c; the shape of the characteristic bands and their wavenumbers were not changed after the thermal cycling test, and the micromorphology of SMA-g-PEG (Figure 4i) maintained morphological stability after the 1000th thermal cycle. The results suggest that the S-SPCM had good thermal stability and that SMA-g-PEG can be reused multiple times.

**Figure 8:**
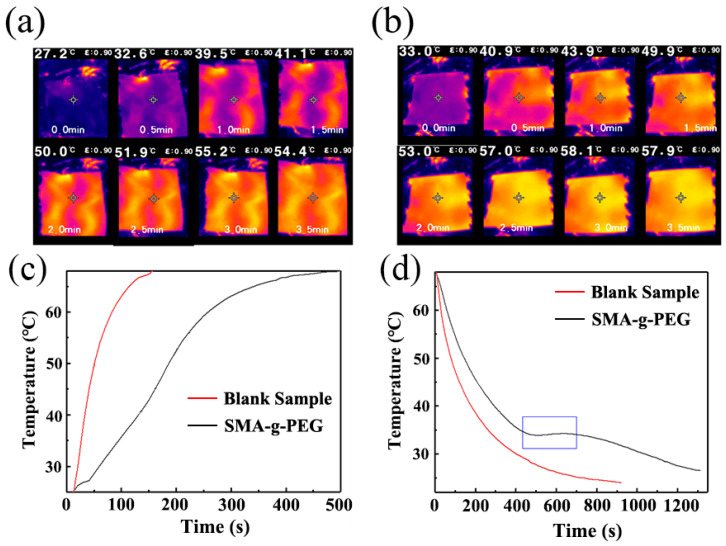
Infrared thermography images of (**a**) SMA-g-PEG in cotton fabric and (**b**) blank cotton (hotplate temperature: 70 °C); (**c**) heating curves (hotplate temperature: 70 °C) and (**d**) cooling curves (hotplate temperature: 20 °C).

To investigate the storage heat ability of S-SPCM in practical applications, SMA-g-PEG (5 g) and a blank sample were filled into a homemade cotton fabric (5 cm × 5 cm) and put on hotplate at 70° C, where infrared thermography images were recorded every 30 s. The measured curve exhibited a certain lag due to the time required for heat transfer. In Figure 8a, the temperature of SMA-g-PEG (41.1 °C) was lower than that of blank cotton (49.9 °C) at 1.5 min (Figure 8b), indicating that the heat absorption effect of the SMA-g-PEG could slow down the temperature change rate and provide a certain temperature control effect [37]. In Figure 8c, compared to the blank sample, SMA-g-PEG exhibited a significant temperature retardation, with a slowdown observed around 45 °C and a maximum temperature difference of 24.8 °C measured using temperature recorder. This was mainly related to the melting process of SMA-g-PEG. During the cooling process, SMA-g-PEG showed a temperature plateau around 34 °C, and the cooling rate was significantly slower than that of the blank, with a maximum temperature difference of 9.8 °C. This was attributed to the solid–solid phase change in the SMA-g-PEG, which occurred within a temperature range corresponding to the DSC test (Figure 8b). In summary, SAM-g-PEG could be used in smart clothing to realize instant energy conversion/storage and temperature regulation [17].

## 4. Conclusions

In this study, SMA was firstly synthesized. PEG was then grafted via bulk-suspension polymerization with PVA as a suspending agent, thus obtaining SMA-g-PEG phase change microspheres. A raw material ratio of 1:1, a reaction time of 6.5 h, a reaction temperature of 70 °C, and a stirring rate of 250 rpm were the optimum reaction conditions for SMA-g-PEG preparation. PEG was successfully fixed, and the grafting rate was 56.2%, benefiting the intrinsic crystalline behavior, thus significantly advancing the phase change behavior. Herein, SMA-g-PEG has an outstanding melting enthalpy of 79.3 J/g and latent heat of SMA-g-PEG after 1000 thermal cycles, with slight changes. On the other hand, SMA-g-PEG was a microsphere with a smooth surface that had a particle size in a range of 100–150 μm, and the powdery product could be used directly. We performed chemical titration to measure the grafting rate of PEG, which was found to be 56.2%. Notably, this study is the first to demonstrate a quantitative relationship between the grafting rate and enthalpy in PEG-based PCMs. Additionally, SMA-g-PEG exhibited good phase change properties in simulated phase change textile experiments. This work could open an avenue to fabricate easily synthesized and stable phase change materials for smart textiles, insulation materials, and for the thermal management of electronic devices.

## Figures and Tables

**Table 1:** Parameters for the synthesis experiment.

Sample	PS: PEG =mol: mol	Stirring Rate (rpm)	Reaction Time (h)	Reaction Temperature (°C)
A	0.5:1/1:1/2:1 yield (%):45.2/54.1/48.9	250	6.5	70
B	1:1	200/250/300 yield (%):42.9/54.1/10.9	6.5	70
C	1:1	250	5/6/6.5/7/7.5 yield (%):38.6/39.9/54.1/5.9/1.0	70
D	1:1	250	6.5	60/65/70/75/80 yield (%):20.7/36.4/54.1/37.1/20.3

**Table 2:** DSC data for the preparation of SMA-g-PEG under different influencing factors.

		Melting			Solidifying		Yield (%)	Grafting Rate (%)
	Onset Temperature (°C)	Peak Temperature (°C)	Latent Heat (J/g)	Onset Temperature (°C)	Peak Temperature (°C)	Latent Heat (J/g)		
Sample A: PS: PEG (mol: mol)								
0.5:1	43.9	49.7	47.3	32.4	29.1	44.5	45.2	29.5
1:1	42.1	48.9	79.3	29.6	24.7	76.9	54.1	52.5
2:1	41.9	47.3	66.2	33.1	28.3	65.7	48.9	43.5
Sample B: Stirring rate								
200 rpm	38.7	47.5	54.3	29.2	25.6	54.1	42.9	35.8
250 rpm	42.1	48.9	79.3	29.6	24.7	76.9	54.1	52.5
300 rpm	53.5	56.3	6.3	31.2	27.3	6.0	10.9	4.0
Sample C: Time								
5 h	39.3	47.0	49.3	33.7	29.6	48.7	38.6	32.2
6 h	41.2	50.2	57.0	33.5	29.8	54.3	39.9	35.9
6.5 h	42.1	48.9	79.3	29.6	24.7	76.9	54.1	52.5
7 h	41.2	45.3	10.8	28.9	25.8	10.6	5.9	7.0
7.5 h	42.4	46.3	4.8	29.9	26.0	4.7	1.0	3.1
Sample D: Temperature								
60 °C	41.3	49.9	40.0	33.9	29.5	39.7	20.7	26.3
65 °C	43.3	48.6	50.0	34.3	29.6	49.9	36.4	33.0
70 °C	42.1	48.9	79.3	29.6	24.7	76.9	54.1	52.5
75 °C	40.3	47.2	66.4	34.2	29.5	66.3	37.1	43.9
80 °C	42.0	47.4	24.1	30.9	27.3	23.9	20.3	15.8

**Table 3:** DSC data of PEG, SMA, SMA-g-PEG, and SMA-g-PEG after 1000th thermal cycle.

Sample	Melting			Solidifying		
	Onset Temperature (°C)	Peak Temperature (°C)	Latent Heat (J/g)	Onset Temperature (°C)	Peak Temperature (°C)	Latent Heat (J/g)
PEG	37.5	52.0	151.1	32.7	32.5	145.2
SMA	27.9	28.5	0.2	0	0	0
SMA-g-PEG	36.5	48.9	79.3	39.6	24.7	76.9
SMA-g-PEG after 1000th cycles	36.2	49.6	65.7	38.9	24.5	63.9

## Data Availability

The data presented in this study are available on request from the corresponding author.

## Abbreviations

PEG	polyethylene glycol
SMA	polystyrene/maleic anhydride
SMA-g-PEG	polystyrene/maleic anhydride grafted polyethylene glycol
S-SPCM	solid–solid phase change material
PCMs	phase change materials
PS	polystyrene
MAH	maleic anhydride
PVA	poly (vinyl alcohol)
AIBN	2-methylpropionitrile
KOH	potassium hydroxide
